# Mechanism of Water Dynamics in Hyaluronic Dermal Fillers Revealed by Nuclear Magnetic Resonance Relaxometry

**DOI:** 10.1002/cphc.201900761

**Published:** 2019-10-17

**Authors:** Danuta Kruk, Pawel Rochowski, Elzbieta Masiewicz, Slawomir Wilczynski, Milosz Wojciechowski, Lionel M. Broche, David J. Lurie

**Affiliations:** ^1^ Faculty of Mathematics and Computer Science University of Warmia & Mazury in Olsztyn Słoneczna 54 10-710 Olsztyn Poland; ^2^ Department of Basic Biomedical Science School of Pharmacy Medical University of Silesia in Katowice Kasztanowa 3 41-200 Sosnowiec Poland; ^3^ Bio-Medical Physics School of Medicine Medical Sciences & Nutrition University of Aberdeen Foresterhill Aberdeen AB25 2ZD, Scotland United Kingdom; ^4^ Current affiliation:Faculty of Mathematics, Physics and Informatics Gdansk University Wita Stwosza 57 80-308 Gdansk Poland

**Keywords:** diffusion, gels, molecular dynamics, nuclear magnetic resonance, relaxation

## Abstract

^1^H spin−lattice nuclear magnetic resonance relaxation experiments were performed for five kinds of dermal fillers based on hyaluronic acid. The relaxation data were collected over a broad frequency range between 4 kHz and 40 MHz, at body temperature. Thanks to the frequency range encompassing four orders of magnitude, the dynamics of water confined in the polymeric matrix was revealed. It is demonstrated that translation diffusion of the confined water molecules exhibits a two‐dimensional character and the diffusion process is slower than diffusion in bulk water by 3–4 orders of magnitude. As far as rotational dynamics of the confined water is concerned, it is shown that in all cases there is a water pool characterized by a rotational correlation time of about 4×10^−9^ s. In some of the dermal fillers a fraction of the confined water (about 10 %) forms a pool that exhibits considerably slower (by an order of magnitude) rotational dynamics. In addition, the water binding capacity of the dermal fillers was quantitatively compared.

## Graphical abstract

**Mechanism of water diffusion**: Profiting from the potential of NMR relaxometry to reveal mechanism of molecular motion in condensed matter, we demonstrate the 2D character of the translation diffusion of water in hyaluronic acid gels. The diffusion process is slower than diffusion in bulk water by 3–4 orders of magnitude.

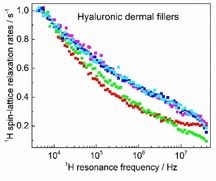

## 1 Introduction

Nuclear magnetic resonance (NMR) relaxometry is one of the most powerful methods of investigating dynamics in condensed matter.^1^, ^2^, ^3^ Classical NMR experiments are performed at a single magnetic field (resonance frequency). NMR relaxometry offers the opportunity to perform NMR relaxation experiments in a broad range of magnetic fields (resonance frequencies).^1^, ^2^, ^3^, ^4^, ^5^ This implies that one can probe, in a single experiment, molecular dynamics in the time scale of 10^−3^ s–10^−9^ s. The unique advantage of NMR relaxometry lies, however, in the ability of revealing not only the time scale of the motion, but also its mechanism;^1^, ^2^, ^6^, ^7^, ^8^, ^9^, ^10^, ^11^, ^12^, ^13^, ^14^, ^15^ the key to this ability is as follows:

Protons (^1^H nuclei) placed in an external magnetic field can assume two quantum states characterized by the magnetic spin quantum number m=±1/2 that correspond to the parallel and anti‐parallel orientations of the ^1^H magnetic moment (spin) with respect to the magnetic field. The energies associated with the two states differ and hence, according to the Boltzmann distribution, the probabilities of occupying the states are different. As a result of the unequal populations of the states, an effective magnetization is created in a system containing many hydrogen atoms (^1^H nuclei). When the external magnetic field is changed, the magnetization evolves in time to the new equilibrium state determined by the Boltzmann distribution, requiring re‐population of the two quantum states. This is possible due to the fact that the magnetic moments (spins) of protons interact not only with the external magnetic field but also with each other. The mutual magnetic dipole‐dipole interactions fluctuate in time as a result of molecular (atomic) motion. The probability of the spin transitions depends on the strength (amplitude) of the dipole‐dipole interactions, the time scale of their fluctuations and the mechanism of the fluctuations. In consequence, the spin‐lattice relaxation rate, characterizing the speed of the magnetization evolution towards its equilibrium value, is given as a linear combination of spectral density functions that are Fourier transforms of corresponding time‐correlation functions associated with the dynamics causing the fluctuations and hence the relaxation. Mathematical forms of the spectral density functions are significantly different depending on the mechanism of the motion:^1^, ^2^, ^6^, ^7^, ^8^, ^9^, ^10^, ^11^, ^12^, ^13^, ^14^, ^15^ rotation, translation of different dimensionality, heterogenous dynamics, etc. The shape of a relaxation dispersion profile (spin‐lattice relaxation rate versus the resonance frequency) is thus a fingerprint of the mechanism of the molecular motion. This potential of NMR relaxometry has been widely exploited for liquids and polymers.^1^, ^3^ However, as far as the translation diffusion is concerned, the process is in most cases three‐dimensional (3D). For liquids in bulk 3D diffusion is to be expected; a reduced dimensionality requires some kind of confinement. 1D diffusion of water in nanotubes has been clearly observed.^9^ It has also been postulated that liquids in porous matrices exhibit two‐dimensional diffusion in the vicinity of the confining walls.^2^, ^16^, ^17^ However, in this case also an alternative interpretation has been proposed,^18^ showing that the diffusion process can be interpreted as still being of 3D character. This indicates that 2D dynamics in soft matter is rather exceptional. In this work, it has been shown that water diffusion in some hyaluronic compounds used as dermal filler is 2D in character.

Hyaluronic acid is one of the main components of the https://en.wikipedia.org/wiki/Extracellular_matrix. It significantly contributes to cell proliferation and migration. Hyaluronic acid is a major component of skin, where it is involved in tissue repair and regeneration.^19^, ^20^, ^21^, ^22^, ^23^, ^24^, ^25^, ^26^, ^27^ It consists of alternately placed monomers of D‐glucuronic acid and N‐acetyl‐D‐glucosamine linked by β‐glycosidic bonds.^23^ At physiological pH, hyaluronic acid occurs primarily in the form of sodium salt and has the ability to bind water. For medical use hyaluronic acid is often subjected to a cross‐linking process that improves its rheological properties.^23^, ^27^ The crosslinking is based on modifications of the carboxyl and hydroxyl groups in order to create connections between polymer chains. If the crosslinking process involves binding together the whole polymer chains, a gel with high density and stiffness is obtained, whereas when only side polymer chain is cross‐linked, a softer, more fluid gel is produced.^23^, ^27^ Hyaluronic acid combined with water (for instance placed in in aqueous environment, *e. g*. in tissues) swells, forming gels which can be used, among other applications, for skin treatments in the form of dermal fillers applied to restore volume of skin and subcutaneous tissue lost for various reasons. The water binding involves formation of hydrogen bonds with the hydrophilic groups of hyaluronic acid: N‐acetyl and carboxyl groups.^28^, ^29^ The water binding capacity, the time‐scale and mechanism of water dynamics in the hyaluronic acid gels are factors determining the medical performance of dermal fillers. NMR relaxometry is a unique method of studying these systems, due to its ability to give insight into the dynamical properties of water confined in the gel matrices.

In consequence, this work has two intertwined goals. The first one is to reveal the mechanisms of water mobility in dermal filler. The second is to demonstrate the ability of NMR relaxometry to unambiguously identify the mechanism of water diffusion, with the purpose to exploit it for revealing possible differences in water dynamics in healthy and pathological tissues.

## 2 Theory

^1^H spin‐lattice relaxation originates from ^1^H‐^1^H magnetic dipole‐dipole interactions. In the present case, taking into account the prevailing water content, the relaxation reflects dynamics of different pools of water in the system. Generally, one can expect a least two pools of water: This implies that the overall ^1^H spin‐lattice relaxation rate, $R_{1}\left(\omega \right)$ , is given as [Eq. (1)]:

(1) $$R_{1}\left(\omega \right)=R_{1}^{conf}\left(\omega \right)+R_{1}^{bulk}\left(\omega \right)$$

where $R_{1}^{conf}\left(\omega \right)$ and $R_{1}^{bulk}\left(\omega \right)$ denote the relaxation contributions associated with the confined and free pools of water, while ω is the ^1^H resonance frequency in angular frequency units. It is important to stress that the pools of water should not be treated as “isolated fractions” – there are exchange processes at a high rate between the bulk and confined states. As far as the pool of confined water is concerned, the relaxation rate, $R_{1}^{conf}\left(\omega \right)$ , stems from intermolecular and intramolecular ^1^H‐^1^H dipole‐dipole interactions modulated by translational and rotational dynamics of water molecules, respectively. Thus, one can rewrite Equation (1) as [Eq. (2)]:

(2) $$R_{1}\left(\omega \right)=R_{1}^{conf,\mathrm{inter}}\left(\omega \right)+R_{1}^{conf,\mathrm{intra}}\left(\omega \right)+A$$

where $R_{1}^{conf,\mathrm{inter}}\left(\omega \right)$ and $R_{1}^{conf,\mathrm{intra}}\left(\omega \right)$ denote the intermolecular and intramolecular relaxation contributions, respectively, while $A=R_{1}^{bulk}\left(\omega \right)$ denotes the relaxation rate of bulk water. The last term is frequency independent as a result of the fast dynamics of bulk water. According to spin relaxation theory, the relaxation rates are given as linear combinations of spectral density functions characterizing the dynamical process leading to the fluctuations of the corresponding dipole‐dipole interactions and hence the relaxation, i. e. [Eq. (3)]:^1^, ^2^, ^3^, ^30^, ^31^, ^32^, ^33^, ^34^

(3) $$R_{1}^{conf,\mathrm{inter}/\mathrm{intra}}\left(\omega \right)=C^{\mathrm{inter}/\mathrm{intra}}\left[J^{\mathrm{inter}/\mathrm{intra}}\left(\omega \right)+4J^{\mathrm{inter}/\mathrm{intra}}\left(2\omega \right)\right]$$

where $C^{\mathrm{inter}/\mathrm{intra}}$ denote the dipolar relaxation constants for the intermolecular and intramolecular relaxation contributions, respectively. The spectral density function, $J^{\mathrm{intra}}\left(\omega \right)$ , takes the form [Eq. (4)]:^1^, ^2^, ^3^, ^30^, ^31^, ^32^, ^35^

(4) $$J^{\mathrm{intra}}\left(\omega \right)=\frac{\tau_{rot}}{1+{\left(\omega \tau_{rot}\right)}^{2}}$$

where $\tau_{rot}$ denotes the rotational correlation time characterizing fluctuations of the ^1^H‐^1^H dipole‐dipole coupling in confined water molecules. The form of the spectral density, $J^{\mathrm{inter}}\left(\omega \right)$ , associated with translation diffusion of the confined water molecules depends on the dimensionality of the diffusion. Anticipating the results, for 2D dynamics the spectral density function yields [Eq. (5)]:^8^, ^10^

(5) $$J^{\mathrm{inter}}\left(\omega \right)=\tau_{trans}\ln\left[1+1/\;{\left(\omega \tau_{trans}\right)}^{2}\right]$$

where $\tau_{trans}$ denotes the translational correlation time. In the low frequency range, when $\omega \tau_{trans}\ll 1$ , Equation (5) converges to $J^{\mathrm{inter}}\left(\omega \right)\propto -\tau_{trans}\ln\left(\omega \tau_{trans}\right)$ which means that the relaxation rate shows a linear dependence on lnω . Eventually, after subtracting the contribution to the overall relaxation originating from bulk water, the relaxation rate $R_{1}\left(\omega \right)$ is given (under the assumption of 2D translation diffusion of the confined water) as [Eq. (6)]:

(6) $$R_{1}\left(\omega \right)=C^{\mathrm{inter}}\tau_{trans}\left[\begin{array}{c} \ln\left(1+1/{\left(\omega \tau_{trans}\right)}^{2}\right)+\;\\ 4\ln\left(1+1/{\left(2\omega \tau_{trans}\right)}^{2}\right)\; \end{array}\right]+\;\;\;\;\;\;\;\;\;\;\;\;\;\;\;\;\;\;\;\;\;\;\;\;\;C^{\mathrm{intra}}\left(\frac{\tau_{rot}}{1+{\left(\omega \tau_{rot}\right)}^{2}}+\frac{4\tau_{rot}}{1+{\left(2\omega \tau_{rot}\right)}^{2}}\right)+A$$

 

## 3 Results and Discussion

Water dynamics in five kinds of dermal filler has been investigated by means of NMR relaxometry. The compounds are labelled as DF1–DF5; their properties are summarized in Table 1. The dermal fillers were purchased from: DF1 (Amalian balance) – S&V Technologies GmbH; DF2 (Princess) – Croma; DF3 (Neauvia organic stimulate) ‐ EUmaterials S.M.; DF4 (Restylane Skinboosters Vital) – Q‐Med AB and DF5 (Skin Rejuvenation Solution, SRS – Hyaluronic Acid 3.5) – SRS International. More comprehensive information is commercially sensitive and is not provided by the suppliers. Density of the compounds is close to 1 g/ml.

**Table 1:** Composition and properties of the investigated dermal fillers.

Compound id.	Name	Chemical composition	Properties
DF1	Amalian balance	hyaluronic acid, 12 mg/ml	non‐crosslinked
DF2	Princess	sodium hyaluronate, 18 mg/ml glycerol 20 mg/ml, phosphate/citrate buffer solution	viscoelastic
DF3	Neauvia organic stimulate	sodium hyaluronate, 26 mg/ml hydroxylapatite <1 %	viscoelastic, stabilized hyaluronic acid, sodium hyaluronate from *Bacillus subtilis*
DF4	Restylane Skinboosters Vital	hyaluronic acid, 20 mg/ml phosphate buffered saline	stabilized hyaluronic acid of non‐animal origin
DF5	Skin Rejuvenation Solution	sodium hyaluronate, 35 mg/ml sodium methylparaben	–

**Figure 1:**
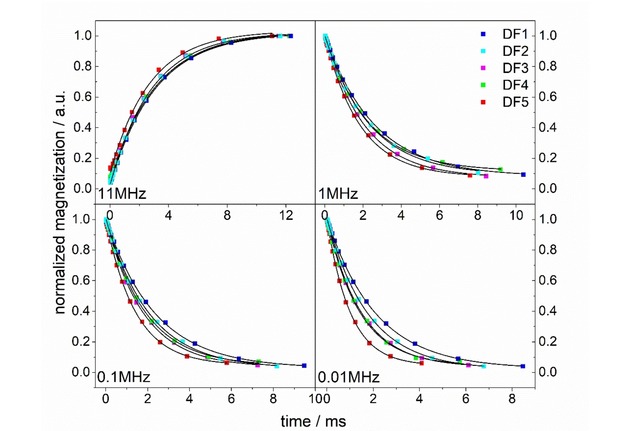
^1^H magnetization versus time for dermal filler labeled as DF1–DF5 for different resonance frequencies.

^1^H spin‐lattice relaxation studies have been performed for these compounds at body temperature (37 °C) in the frequency range spanning four orders of magnitude, from 4 kHz to 40 MHz. The relaxation process was found to be single‐exponential for all samples at all magnetic fields; examples of ^1^H magnetization curves recorded versus time are shown in Figure 1.

**Figure 2:**
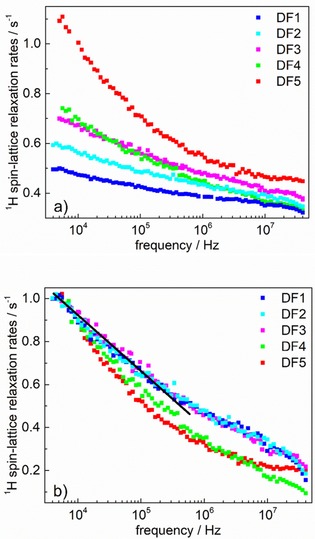
^1^H spin−lattice relaxation dispersion profiles for hydrogels: a) direct result of the experiment, b) after subtracting bulk water ^1^H spin−lattice relaxation rate (0.29 s^−1^) and normalizing to unity at the low frequency limit. Solid line indicates a linear dependence of the ^1^H spin−lattice relaxation rate on lnω .

The ^1^H spin‐lattice relaxation dispersion data for the set of dermal fillers, DF1–DF5, is shown in Figure 2a. The first impression is that the shapes of the relaxation dispersion profiles for the systems are quite different. However, after subtracting from the relaxation data the relaxation contribution originating from bulk water, A=0.29 s^−1^, and normalizing the remaining relaxation rates to unity at the lowest frequency, the graphs shown in Figure 2b were obtained. The normalization required division of the data by: 0.2 (DF1), 0.3 (DF2), 0.4 (DF3), 0.45 (DF4), and 0.8 (DF5). One can clearly see from Figure 2b that the relaxation data for DF1, DF2 and DF3 overlap, defining a “master curve ” from which the data for DF4 and DF5 deviate. Moreover, the normalized relaxation rates for DF1, DF2 and DF3 follow at low frequencies a linear dependence on lnω over two frequency decades; such a dependence is a marker of 2D translation diffusion. One might attribute the diffusion in the reduced dimensionality (2D) to a translation motion along the hyaluronic chains. The 2D diffusion suggests that the chains form locally flat surfaces.

**Figure 3:**
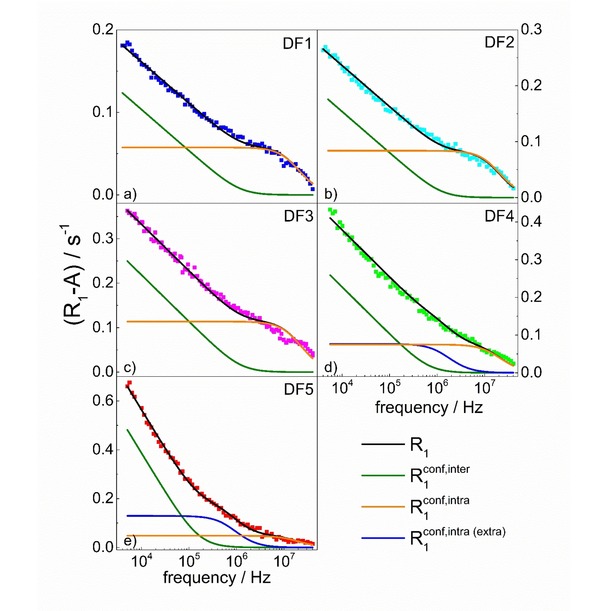
^1^H spin−lattice relaxation dispersion profiles for DF1–DF5 reproduced by means of Equation (6); black lines – overall fits decomposed into the $R_{1}^{conf,\mathrm{inter}}\left(\omega \right)$ and $R_{1}^{conf,\mathrm{intra}}\left(\omega \right)$ contributions for DF1, DF2 and DF3, including the additional term$R_{1}^{conf,\mathrm{intra}(\mathrm{extra})}\left(\omega \right)=C^{\mathrm{intra}(\mathrm{extra})}\left(\frac{\tau_{rot(extra)}}{1+{\left(\omega \tau_{rot(extra)}\right)}^{2}}+\frac{4\tau_{rot(extra)}}{1+{\left(2\omega \tau_{rot(extra)}\right)}^{2}}\right)$ for DF4 and DF5. The frequency independent term, A , has been subtracted from the experimental data. The translation diffusion coefficients of water molecules in the confinement, *D_trans_*, have been estimated from the $\tau_{trans}$ values; they are also included in Table 2.

As the relaxation rates overlap, one can easily conclude that the correlation times $\tau_{trans}$ and $\tau_{rot}$ for DF1, DF2 and DF3 are very similar, while the dipolar relaxation constants scale by factors which can readily be estimated from the normalization procedure. The analysis of the relaxation data for DF1, DF2 and DF3 and the decomposition into the $R_{1}^{conf,\mathrm{intra}}\left(\omega \right)$ and $R_{1}^{conf,\mathrm{inter}}\left(\omega \right)$ contributions is shown in Figure 3a–c. The results are collected in Table 1. The translational correlation time is defined as: $\tau_{trans}=\frac{d^{2}}{D_{trans}}$ ,^6^, ^7^, ^14^, ^15^ where *d* is referred to as distance of closest approach and can be estimated by the diameter of water molecule (2.75 Å).

^1^H water relaxation rates in confinement are proportional (after subtracting the contribution to the overall relaxation from bulk water) to the fraction of bound water molecules, providing the exchange lifetime, $\tau_{ex}$ , between the confined and bulk water pools is sufficiently short, so it can be omitted in the dominator of Equation (7):^38^, ^39^

(7) $$R_{1}\left(\omega \right)-A=\frac{Pq}{{\left[R_{1(norm)}^{conf,\mathrm{intra}}\left(\omega \right)\right]}^{-1}+\tau_{ex}}+R_{1}^{conf,\mathrm{inter}}\left(\omega \right)$$

The *P* and *q* quantities denote the mole fraction of water protons in the bound position (*P*=*c*/55.6, where *c* denotes concentration in mole of the molecules building the confinement–hyaluronic acid, in this case) and the coordination number, respectively.

The coordination number (number of protons bounded to a single confining molecule) can be here considered as a measure of water binding capacity of the dermal fillers. The relaxation rate, $R_{1(norm)}^{conf,\mathrm{intra}}\left(\omega \right)$ , can be treated as $R_{1}\left(\omega \right)$ for *Pq*=1. Comparing Equation (7) with Equation (2) and Equation (3) one can easily see that Equation (2) takes the form of Equation (7), when $C^{\mathrm{intra}}$ includes the *Pq* product. Analogously, the intermolecular dipolar relaxation constant, $C^{\mathrm{inter}}$ , includes the number of confined water molecules (number of protons) per unit volume, i. e. it is proportional to the *Pq* value. The ratios between the dipolar relaxation constants listed in Table 2 ($\frac{C^{\mathrm{inter}}}{C^{\mathrm{inter}}\left(DF_{1}\right)}$ and $\frac{C^{\mathrm{intra}}}{C^{\mathrm{intra}}\left(DF_{1}\right)}$ ) are in agreement with the ratios between the hyaluronate contents in DF1, DF2 and DF3, that yields: 1.2 : 1.8 : 2.6 (i. e. 1 : 1.5 : 2.16). This implies that the water binding capacity of this group of dermal fillers is very similar. The translational diffusion coefficient *D_trans_* is about 1×10^−12^ m^2^/s and it is about 3×10^3^ times smaller than the diffusion coefficient of bulk water.^40^ Strictly speaking, *D_trans_* denotes the relative diffusion coefficient of the confined and confining molecules, but one can expect that the water diffusion is much faster than the diffusion of the polymer. The rotational correlation time of the confined water molecules, $\tau_{rot}$ , is of the order of 4×10^−9^ s, yielding the $\frac{\tau_{trans}}{\tau_{rot}}$ ratio of 17–21. The ratio is typical for liquids.^3^ Eventually, the frequency independent term, *A*, yielding (0.32–0.33) s^−1^, is close to the relaxation rate of bulk water.

**Table 2:** Parameters obtained from analysis of ^1^H spin‐lattice relaxation data for dermal fillers.

Compound id.	$C^{\mathrm{inter}}$ [Hz^2^]	$\tau_{trans}$ [s]	$C^{\mathrm{intra}}$ [Hz^2^]	$\tau_{rot}$ [s]	A [s^−1^]	$D_{trans}$ [m^2^/s]	$\frac{\tau_{trans}}{\tau_{rot}}$	$\frac{C^{\mathrm{inter}}}{C^{\mathrm{inter}}\left(DF1\right)}$	$\frac{C^{\mathrm{intra}}}{C^{\mathrm{intra}}\left(DF1\right)}$	St. dev. [%]
DF1	2.79×10^4^	7.78×10^−8^	2.72×10^6^	4.23×10^−9^	0.32	9.72×10^−13^	18	1	1	0.73
DF2	4.29×10^4^	7.46×10^−8^	3.98×10^6^	4.21×10^−9^	0.33	1.01×10^−12^	17	1.54	1.46	1.06
DF3	6.05×10^4^	7.53×10^−8^	5.89×10^6^	3.86×10^−9^	0.34	1.04×10^−12^	21	2.17	2.17	1.46
Compound id.	$C^{\mathrm{inter}}$ [Hz^2^	$\tau_{trans}$ [s]	$C^{\mathrm{intra}}$ [Hz^2^]	$\tau_{rot}$ [s]	A [s^−1^]	$D_{trans}$ [m^2^/s]	$\frac{\tau_{trans}}{\tau_{rot}}$	$\frac{C^{\mathrm{inter}}}{C^{\mathrm{inter}}\left(DF1\right)}$	$\frac{C^{\mathrm{intra}}}{C^{\mathrm{intra}}\left(DF1\right)}$	St. dev. [%]
			Cintra(extra) [Hz^2^]	$\tau_{rot(extra)}$ [s]			$\frac{\tau_{trans}}{\tau_{rot(extra)}}$		Cintra(extra)Cintra(DF1)	
DF4	3.92×10^4^	1.38×10^−7^	3.62×10^6^	4.13×10^−9^	0.31	5.48×10^−13^	33	1.41	1.33	1.68
			3.09×10^5^	4.96×10^−8^			2.8		0.11	
DF5	2.45×10^4^	5.69×10^−7^	2.19×10^6^	4.37×10^−9^	0.43	1.33×10^−13^	130	0.88	0.81	1.16
			2.79×10^5^	9.28×10^−8^			6.1		0.1	

Diffusion of water confined in DF4 and DF 5 is also of 2D character. However, as the translation dynamics in DF4 and DF5 is slower, the linear dependence is limited to lower frequencies. However, the rotational dynamics is more complex. The concept of a single pool of confined water breaks down as the relaxation data cannot be interpreted in terms of Equation (6). The data indicate the presence of two pools of confined water that undergo rotational dynamics on different time scales, although their translation dynamics is the same. This requires extending Equation (6) to the form [Eq. (8)]:

(8) $$R_{1}\left(\omega \right)=C^{\mathrm{inter}}\tau_{trans}[\ln\left(1+1/{\left(\omega \tau_{trans}\right)}^{2}\right)+4\ln(1+1/{\left(2\omega \tau_{trans}\right)}^{2})]+C^{\mathrm{intra}}\left(\frac{\tau_{rot}}{1+{\left(\omega \tau_{rot}\right)}^{2}}+\frac{4\tau_{rot}}{1+{\left(2\omega \tau_{rot}\right)}^{2}}\right)+C^{\mathrm{intra}(\mathrm{extra})}\left(\frac{\tau_{rot(extra)}}{1+{\left(\omega \tau_{rot(extra)}\right)}^{2}}+\frac{4\tau_{rot(extra)}}{1+{\left(2\omega \tau_{rot(extra)}\right)}^{2}}\right)+A$$

Figure 3d,e shows the data analysis in term of Equation (7). The obtained parameters are included in Table 2. It has been assumed that the exchange lifetime for both pools of confined water (exhibiting different rotational dynamics) are much faster than the corresponding relaxation contributions, and both the dipolar relaxation constants, $C^{\mathrm{intra}}$ and $C^{\mathrm{intra}(\mathrm{extra})}$ include the *Pq* and *Pq*_*(extra)*_ factors, respectively. The translation diffusion coefficient for DF4 has a value smaller than the values for DF1, DF2, DF3 by a factor of about 5. The rotational correlation time for the dominating pool of confined water is of about 4×10^−9^ s, in analogy to the DF1, DF2, DF3 group. The resulting $\frac{\tau_{trans}}{\tau_{rot}}$ ratio (33) is still in the range reported for several liquids.^3^ The rotational dynamics of the second (extra) pool of water molecules is by an order of magnitude slower (the corresponding rotational correlation time,$\tau_{rot(extra)}$ , yields about 5×10^−8^ s). From the ratios $\frac{C^{\mathrm{inter}}}{C^{\mathrm{inter}}\left(DF_{1}\right)}$ =1.41 and $\frac{C^{\mathrm{intra}}+C^{\mathrm{intra}(\mathrm{extra})}}{C^{\mathrm{intra}}\left(DF_{1}\right)}$ =1.44 one might conclude that the water binding capacity is similar than for to the DF1, DF2, DF3 group. It should be noted that the ratios for the intermolecular and intramolecular dipolar relaxation constants should be the same, as the whole fraction of confinement water participates in translational and rotational dynamics; the small differences stem from the fitting procedure keeping both of them independent. Comparing the $C^{\mathrm{intra}}$ and $C^{\mathrm{intra}(\mathrm{extra})}$ dipolar relaxation constants one can determine that that the pool of water molecules undergoing slower rotational motion includes about 10 % of the confined water. Eventually, one should mention that the *A* term for DF4 is close to the relaxation rate of bulk water.The translation diffusion of water confined in DF5 is an order of magnitude slower than that of water in DF1, DF2, DF3. Again, the rotational correlation time for the dominating pool of confined water is of about 4×10^−9^ s. However, in this case the rotational dynamics of the second pool is even slower; the ratio $\frac{\tau_{rot(extra)}}{\tau_{rot}}$ reaches about 20. The $\frac{C^{\mathrm{inter}}}{C^{\mathrm{inter}}\left(DF_{1}\right)}$ =0.88 and $\frac{C^{\mathrm{intra}}+C^{\mathrm{intra}(\mathrm{extra})}}{C^{\mathrm{intra}}\left(DF_{1}\right)}$ =0.91 values show that the water binding capacity for DF5 is considerably lower; following the hyaluronate concentration it should yield 2.92 (i. e. about a factor of 3 more). In analogy to DF4 the pool of water molecules undergoing slower rotational dynamics constitutes about 10 % of the confined water. The *A* term for DF5 is about a factor of 1.5 larger than the relaxation rate of bulk water, but this does not change the overall scenario of the water dynamics.

## 4 Conclusions

^1^H NMR relaxometry has been exploited to enquire into the mechanism and time scale of water dynamics in selected hyaluronic dermal fillers. It has been found that in all cases the translation diffusion of the confined water is of 2D character and the corresponding diffusion coefficients are smaller than for water in bulk by 3–4 orders of magnitude. Interestingly, for dermal fillers labelled as DF1, DF2 and DF3, the translation diffusion coefficients are almost the same. Following the line of similarities, it has been demonstrated that in all cases there is a pool of the confined water molecules undergoing rotational dynamics characterized (in a very good approximation) by a correlation time of 4×10^−9^ s. For DF1, DF2 and DF3 the whole fractions of confined water exhibit such rotational dynamics. For DF4 and DF5 about 10 % fractions of the confined water perform rotational motion on a significantly longer (by an order of magnitude) time scale. Moreover, it has been shown that water binding capacity of the dermal fillers DF1, DF2, DF3 and DF4 are very similar, while for DF5 the water binding capacity is about a factor of 3 lower.

Independently of the knowledge about water dynamics in hyaluronic dermal fillers gained from the studies, the results demonstrate the unique ability of NMR relaxometry to reveal in a single experiment not only the time scales of molecular motion in a broad time range, but also the underlying mechanisms of the motional processes, rendering this method a powerful tool for studying dynamics in biological systems.

## Experimental Section

The ^1^H spin‐lattice relaxation measurements were performed using a commercially‐available NMR Fast Field‐Cycling relaxometer (Stelar S.r.l., Mede, Italy, model SPINMASTER FFC2000). The sample temperature was controlled with an accuracy of 0.5 °C. Below 10 MHz the pre‐polarized sequence was used, while above 10 MHz the non‐polarized sequence was applied.^41^

## Conflict of interest

The authors declare no conflict of interest.
